# Feasibility of coding-based Charlson comorbidity index for hospitalized patients in China, a representative developing country

**DOI:** 10.1186/s12913-020-05273-8

**Published:** 2020-05-18

**Authors:** Liyi Mo, Zhen Xie, Guohui Liu, Qiang He, Zhiming Mo, Yanhua Wu, Wenji Wang, Feng Ding, Yuanjiang Liao, Li Hao, Chen Lu, Jin Sun, Libin Xu, Yusheng Zhang, Rizwangul Ghappar, Hongwei Peng, Xiaohong Wei, Jinglie Xie, Yuanhan Chen, Xinling Liang, on behalf of China collaborative study on acute kidney injury

**Affiliations:** 1 Division of Nephrology, Guangdong Provincial People’s Hospital, Guangdong Academy of Medical Sciences, No.106 Zhongshan Road 2, Guangzhou, 510080 Guangdong China; 2 Department of Nephrology, Dongguan People’s Hospital, Dongguan, 523018 China; 3 Department of Dermatology, Sichuan Academy of Medical Sciences & Sichuan Provincial People’s Hospital, Chengdu, 610072 China; 4 Department of Nephrology, Zhejiang Provincial People’s Hospital (People’s Hospital of Hangzhou Medical College), Hangzhou, 310014 China; 5 Division of Nephrology, Shanghai Ninth People’s Hospital, School of Medicine, Shanghai Jiaotong University, Shanghai, 200030 China; 6 Department of Nephrology, Chongqing Ninth People’s Hospital, Chongqing, 400700 China; 7 Department of Nephrology, Second Hospital of Anhui Medical University, Hefei, 230601 China; 8 Department of Nephrology, People’s Hospital of Xinjiang Uygur Autonomous Region, Urumqi, 830001 China; 9 Department of Nephrology, Second Hospital of Jilin University, Changchun, 130022 China; 10 Department of Nephrology, Inner Mongolia People’s Hospital, Hohhot, 010017 China; 11 Second Division of Internal Medicine, Wuhua People’s Hospital, Meizhou, 514400 China; 12 Department of Nephrology, First People’s Hospital of Kashgar, Kashgar, 844000 China; 13 People’s Hospital of Wanning & The First Affiliated Hospital of Chongqing Medical Univesity, Wanning, 571500 China; 14 Department of Nephrology, Chongzuo People’s Hospital, Chongzuo, 844000 China; 15 Division of Nephrology, Guangdong Lufeng People’s Hospital, Lufeng, 516500 China

**Keywords:** ICD-10, Charlson comorbidity index, Diagnosis, Agreement, Discrimination

## Abstract

**Background:**

The Charlson Comorbidity Index (CCI) can be automatically calculated from the International Classification of Disease (ICD) code. However, the feasibility of this transformation has not been acknowledged, particularly in hospitals without a qualified ICD coding system. Here, we investigated the utility of coding-based CCI in China.

**Methods:**

A multi-center, population-based, retrospective observational study was conducted, using a dataset incorporating 2,464,395 adult subjects from 15 hospitals. CCI was calculated using both ICD-10-based and diagnosis-based method, according to the transformation rule reported previously and to the literal description from discharge diagnosis, respectively. A κ coefficient of variation was used as a measure of agreement between the above two methods for each hospital. The discriminative abilities of the two methods were compared using the receiver-of-operating characteristic curve (ROC) for prediction of in-hospital mortality.

**Results:**

Total agreement between the ICD-based and diagnosis-based CCI for each index ranged from 86.1 to 100%, with κ coefficients from 0.210 [95% confidence interval (CI) 0.208–0.212] to 0.932 (95% CI 0.924–0.940). None of the 19 indices of CCI had a κ coefficient > 0.75 in all the hospitals included for study. The area under the curve of ROC for in-hospital mortality of all 15 hospitals was significantly lower for ICD-based than diagnosis-based CCI [0.735 (0.732, 0.739) vs 0.760 (0.757, 0.764)], indicative of more limited discriminative ability of the ICD-based calculation.

**Conclusions:**

CCI calculated using ICD-10 coding did not agree with diagnosis-based CCI. ICD-based CCI displayed diminished discrimination performance in terms of in-hospital mortality, indicating that this method is not promising for CCI scoring in China under the present circumstances.

## Background

The Charlson comorbidity index (CCI) is a scoring system to classify or assign weights to comorbid conditions. The index was initially developed in a small cohort of patients for predicting one-year mortality and tested in another cohort during a 10-year follow-up period [1]. After years of clinical practice, CCI not only facilitated prediction of short- and long-term mortality but could also be utilized to measure disease burden in multiple clinical settings [2, 3].

CCI involves 19 comorbidities, which can be extracted from clinical diagnoses or the corresponding International Classification of Disease (ICD) codes. Compared to the considerable work involved in one-to-one calculations based on diagnosis, CCI can be automatically and quickly calculated using the ICD code [4]. Accordingly, ICD-based CCI is widely used. The most extensively applied version is ICD-10 published in 1993. However, CCI assessed using the ICD-10 code does not completely match that from clinical diagnosis. Accurate reclassification of clinical diagnoses that do not match the ICD-10 code requires the professional clinical knowledge of coders and occasionally clinicians [5]. Due to disagreements between diagnosis and ICD-10 code-based methods, ICD-10 generalization involves long time-periods. National administrative departments in developed countries, such as the Department of Health and Human Services in the United States, are in charge of adaptations of ICD modifications and updates to ensure concordance with diagnosis [6], (https://www.cdc.gov/nchs/data/icd/10cmguidelines_2017_final.pdf). ICD has also been widely applied in developing countries [7–9] but its use in these cases is non-standard. China officially started to use ICD-10 in 2002 and attempted to promote a 6-digit extension code of ICD-10 in 2012. As the world’s largest developing country, China should provide valuable information for the effective implementation of ICD. Previous studies in China disclosed a 4.79–73.08% error rate of coding [10]. Considering the overall heterogenicity and relatively poor coding quality in China, the feasibility of coding-based CCI should be investigated.

The main objective of present study was to ascertain the utility of coding-based CCI through comparison with diagnosis-based CCI.

## Methods

### Study design and data sources

A multi-center, population-based, retrospective observational study was conducted, using the phase 1 dataset of the China Collaborative Study on Acute Kidney Injury, which contains all the literal discharge diagnoses with relative ICD-10 codes and in-hospital death records. This multicenter retrospective observational study was designed to identify novel risk factors of acute kidney injury. The registration number in clinicaltrials.gov is NCT03061786. The study protocol complied with the Declaration of Helsinki and was approved by the Ethics Research Committees of Guangdong General Hospital (GDREC2016327H).

**Fig. 1:**
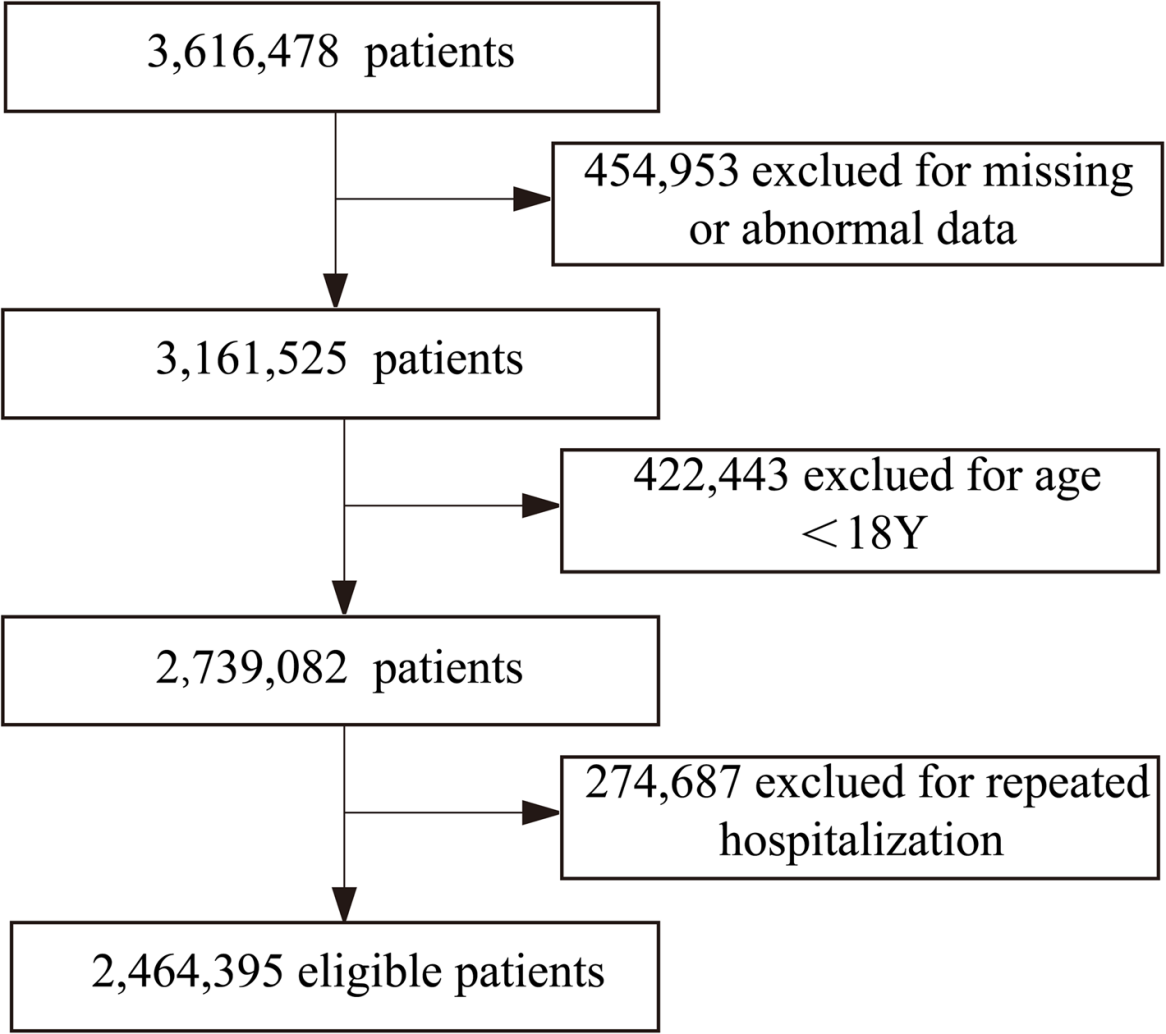
Flow chart of the selected study population

The phase 1 dataset included 3,616,478 adult (18 years or older) admissions in 15 hospitals from January 2012 to December 2016 across 9 provinces in China (Guangdong, Sichuan, Zhejiang, Anhui, Jilin, Shanghai, Chongqing, Inner Mongolia and Xinjiang). Twelve of these were tertiary hospitals and the remaining three were secondary hospitals (Supplementary Table 1). The hospital names were anonymized in reports owing to privacy considerations. The exclusions criteria were as follows: 1) missing or abnormal data (including data of age, hospitalization stay or medical cost); 2) younger than 18 years old; 3) repeated hospitalization (Fig. 1).

### CCI calculation

CCI was calculated using both ICD-10-based and diagnosis-based methods. ICD-10-based CCI was assessed according to the transformation rule reported in previous studies (Supplementary Table 2) [11–13] while diagnosis-based CCI was calculated based on the literal description from discharge diagnosis, regarded as the “gold standard”. Calculations were independently performed by two trained physicians. In cases where the calculations were inconsistent, final classification was made by the research group.

### Statistical analysis

Data with normal distribution are presented as means ± SD and data with non-normal distribution as median values (25th or 75th percentile). Differences between two groups were determined using the independent-samples *t*-test or Mann–Whitney *U* test, as appropriate. Numerical data were evaluated as proportions. Percentage agreement and κ statistic were calculated to evaluate the degree of agreement between ICD-based and diagnosis-based CCI. The κ coefficient of variation (SD/mean × 100%) was applied as a measure of agreement variations among hospitals, with κ coefficient <  0.75 defined as poor agreement. Discrimination abilities of the methods were compared based on the area under the curve of receiver of operating characteristic (AUC of ROC) using R software (Version 1.0.153). Other statistical analyses were undertaken using SPSS version 24.0 (IBM, Armonk, NY, USA). Two-tailed *P* <  0.05 was considered statistically significant.

## Results

### Clinical characteristics of subjects

A total of 2,464,395 subjects were included. Median of the comorbidity number was 1 and ranged from 0 to 10 according to diagnosis-based CCI. The characteristics of the subjects are presented in Table 1.

**Table 1:** Demographic and clinical characteristics

Clinical characteristics	
Age (years)	53 ± 18
Number of men (%)	47.0
Department	
Internal Medicine department (%)	35.0
Surgery department (%)	35.7
Gynecology and Obstetrics (%)	13.4
Emergency department and ICU (%)	5.0
Oncology department (%)	6.0
Others (%)	4.9
Hospital stay (Days)	8 (5,13)
Medical cost (dollars)	1478.2 (858.5, 2808.7)
In-hospital Mortality (%)	0.8

### Comorbidity distributions

According to discharge diagnoses, the comorbidity frequencies of CCI (from high to low) were as follows: cerebrovascular disease, tumor, mild liver disease, diabetes without chronic complication, congestive heart failure, chronic pulmonary disease, peripheral vascular disease, renal disease, metastatic solid tumor, diabetes with chronic complication, myocardial infarction, rheumatologic disease, peptic ulcer disease and hemiplegia (Supplementary Table 3). The other six rare comorbidities with < 1% incidence were lymphoma, moderate or severe liver disease, leukemia, dementia, hemiplegia, and acquired immune deficiency syndrome (AIDS) (Supplementary Table 3).

### Disagreement between ICD-based and diagnosis-based CCI

**Fig. 2:**
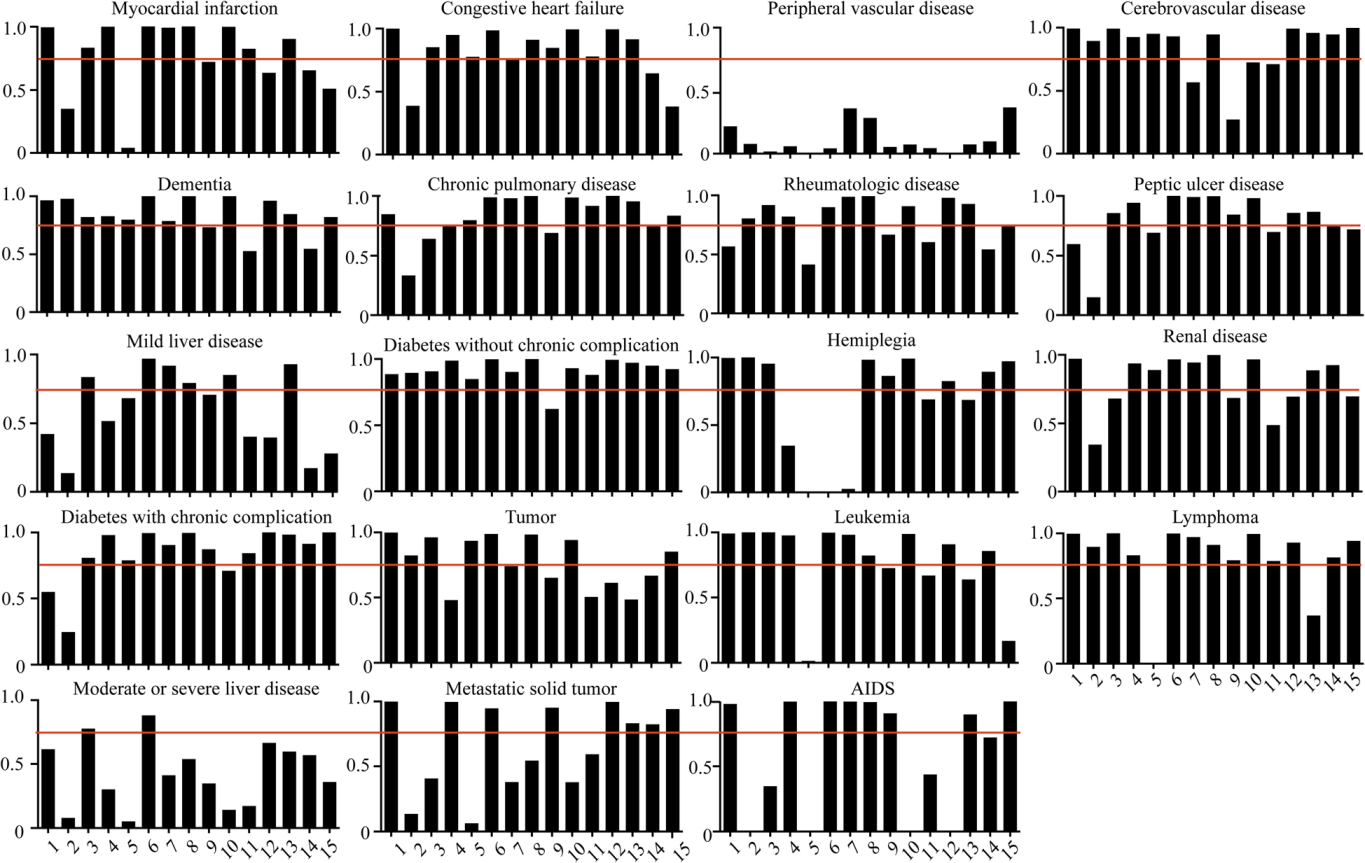
Agreement between the ICD-based and diagnosis-based CCI for each index. The red horizontal line denotes a κ coefficient of 0.75. The Y-axis values denote κ coefficient, which is used as a measure of agreement variation. The red horizontal line denotes a κ coefficient of 0.75

Total agreement between ICD-based and diagnosis-based CCI for each index ranged from 86.1% (κ = 0.210, 95% CI 0.208–0.212) to 100% (κ = 0.932, 95% CI 0.924–0.940) (Table 2). None of the 19 indices had a κ coefficient > 0.75 in all the hospitals examined (Fig. 2). Typically, for all 15 hospitals, low κ coefficients < 0.75 for peripheral vascular disease were obtained, comparable to 13 hospitals for moderate or severe liver disease and 9 hospitals for mild liver disease (Fig. 2).

**Table 2:** Correlation coefficient and κ statistic between ICD-based and diagnosis-based CCI

Items of the Charlson comorbidity scoring system	correlation coefficient	κ(95% CI)	*P* value
Myocardial infarction	0.836	0.826 (0.824, 0.828)	0.001
Congestive heart failure	0.816	0.805 (0.803, 0.807)	0.001
Peripheral vascular disease	0.221	0.210 (0.208, 0.212)	0.001
Cerebrovascular disease	0.897	0.897 (0.897, 0.897)	< 0.001
Dementia	0.907	0.907 (0.903, 0.911)	0.002
Chronic pulmonary disease	0.885	0.883 (0.881, 0.884)	0.001
Rheumatologic disease	0.788	0.770 (0.766, 0.774)	0.002
Peptic ulcer disease	0.814	0.807 (0.803, 0.811)	0.002
Mild liver disease	0.603	0.595 (0.593, 0.597)	0.001
Diabetes without chronic complication	0.935	0.934 (0.934, 0.934)	< 0.001
Hemiplegia	0.401	0.292 (0.284, 0.300)	0.004
Renal disease	0.854	0.851 (0.849, 0.852)	0.002
Diabetes with chronic complication	0.918	0.918 (0.916, 0.920)	0.001
Tumor	0.828	0.821 (0.819, 0.822)	0.001
Leukemia	0.730	0.710 (0.701, 0.716)	0.002
Lymphoma	0.921	0.920 (0.918, 0.922)	0.001
Moderate or severe liver disease	0.465	0.451 (0.445, 0.457)	0.003
Metastatic solid tumor	0.813	0.797 (0.795, 0.799)	0.001
AIDS	0.934	0.932 (0.924, 0.940)	0.004

### Discrimination ability of ICD-based and diagnosis-based CCI for in-hospital death

**Fig. 3:**
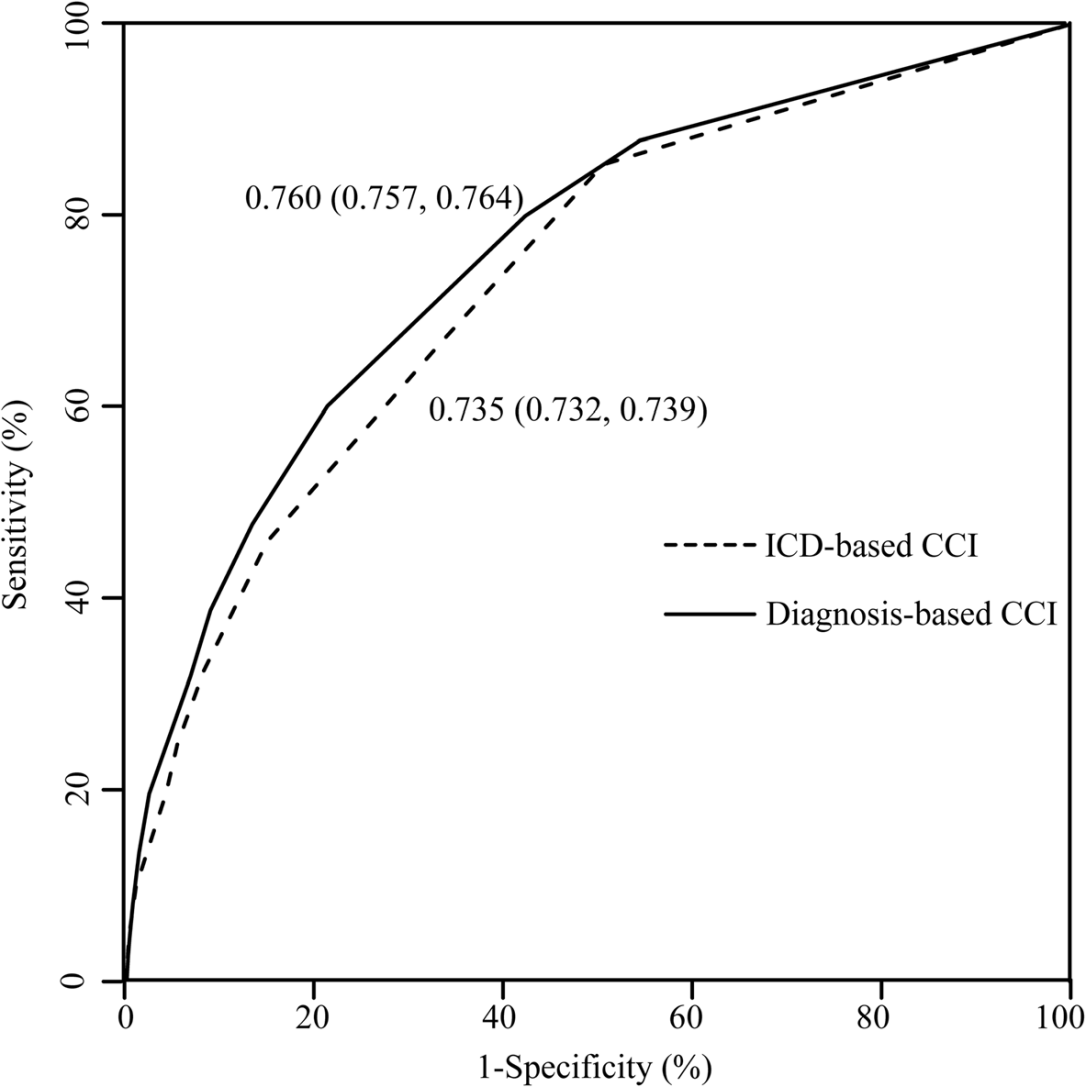
Discriminatory ability of ICD-based and diagnosis-based CCI for in-hospital mortality

We further compared discrimination ability of the two methods with regard to in-hospital mortality of ICD-based and diagnosis-based CCI by calculating AUC of ROC. AUCs of ICD-based CCI ranged from 0.556 (95% CI 0.516, 0.596) to 0.844 (95% CI 0.819, 0.868) and those of diagnosis-based CCI from 0.585 (95% CI 0.562, 0.608) to 0.849 (95% CI 0.817, 0.865). Total AUC was significantly lower for ICD-based CCI relative to diagnosis-based CCI [0.735 (0.732, 0.739) vs 0.760 (0.757, 0.764), *P* <  0.001] in all 15 hospitals (Fig. 3) as well as AUC values from10 individual hospitals (supplementary Table 4). In two hospitals, AUC values for ICD-based CCI were similar to those for diagnosis-based CCI [0.843 (0.819, 0.868) vs 0.849 (0.817, 0.865), *P* = 0.625; 0.713(0.700, 0.725) vs 0.718 (0.705, 0.730), *P* = 0.234]. AUC in one of the above hospitals was also the highest for CCI based on both methods while in three other hospitals, AUCs for ICD-based CCI were higher than those for diagnosis-based CCI [0.739 (0.716, 0.761) vs 0.717 (0.694, 0.740), *P* = 0.011; 0.603 (0.582, 0.625) vs 0.585 (0.562, 0.608), *P =* 0.013; 0.670 (0.652, 0.689) vs 0.657 (0.638, 0.675), *P* <  0.001]. The relatively low AUC values in these three hospitals are indicative of limited value of any type of CCI (supplementary Table 4).

## Discussion

This hospitalized population-based study revealed significant differences in intra-hospital comorbidity distributions [14]. ICD-based CCI did not match corresponding diagnosis-based CCI, particularly for peripheral vascular and liver diseases. None of the 19 indices showed satisfactory agreement (κ coefficient > 0.75) in any of the 15 hospitals examined, reflecting frequent discrepancies. Though the κ coefficient were generally higher than Januel et al. reported in 2003 [15]. Furthermore, ICD-based CCI was associated with lower AUC of ROC for in-hospital mortality than diagnosis-based CCI, indicative of a diminished discrimination performance, consistent with earlier studies [16, 17].

Several factors may contribute to the poor performance of ICD-based CCI, the most important being variable intra-hospital coding qualities. Distinct from American hospitals in which a national standard of ICD-Clinical Modification is adopted, Chinese hospitals modify ICD coding at the individual hospital level. Experienced coding personnel are particularly scarce in China and most are not fully trained [10]. Second, inputted Chinese diagnosis-based ICD coding does not match in a one-to-one manner in some cases, leading to inaccurate classification or even missing an ICD code [18]. Third, the qualities of ICD coding and recording are not comprehensively evaluated. Thus, in hospitals without a qualified coding system, direct application of ICD-based CCI should be avoided.

In addition to the implication of lower discrimination performance of ICD-based CCI, its convenience merits consideration. Notably, in a few hospitals (for example, hospital No. 15), ICD-based CCI displayed discriminative value for in-hospital mortality comparable to that of diagnosis-based CCI. Based on our results, we recommend that in hospitals with or without a qualified coding system, physicians and researchers should be aware of the limitations of CCI involving indices and acknowledge the potential errors of direct adoption of ICD-based CCI. Further validation of these indices is advocated, and standardization of ICD-10 coding remains an urgent task. In the future, national standards, specialized training and transformation software should be implemented to improve the reliability of ICD-based CCI along with the progress of hospital information management.

The large sample size including more than 3 million patients is a major strength of this study. Data were derived from hospital populations and both tertiary and secondary hospitals were included, thus minimizing selection bias. In addition, the hospitals included for study were distributed across various geographical and economic regions in China. Our experiences may therefore be applicable to other developing countries.

## Conclusion

In conclusion, ICD-10 coding-based CCI does not concur with diagnosis-based CCI and is therefore not a promising technique for CCI scoring in China under the present circumstances.

## Supplementary information

**Additional file 1.** Table S1. Geographical and economic information about the hospitals included for study.

**Additional file 2.** Table S2. Previously reported ICD-10 coding for Charlson comorbidity index.

**Additional file 3.** Table S3. Prevalence of comorbidities based on ICD-10 Coding Algorithms and diagnosis at different hospital levels.

**Additional file 4.** Table S4. AUC for in-hospital mortality using ICD-based and diagnosis-based CCI.

## Data Availability

The data that support the findings of this study are available from the corresponding authors but restrictions apply to the availability of these data, which were used under license for the current study, and so are not publicly available. Data are however available from the authors upon reasonable request and with permission of the corresponding authors have obtained in order to freely share the hospital data.

## Abbreviations

AUC: Area under the curve
CCI: Charlson Comorbidity Index
ICD: International Classification of Disease
ROC: Receiver-of-operating characteristic curve
