# Supplementary material for: Feasibility of coding-based Charlson comorbidity index for hospitalized patients in China, a representative developing country
Source: BMC Health Serv Res. 2020 May 18;20:432. doi: 10.1186/s12913-020-05273-8 (PMC7236530; doi:10.1186/s12913-020-05273-8)
Supplement: Supplementary file 1 — Additional file 1. Table S1. Geographical and economic information about the hospitals included for study. [file 12913_2020_5273_MOESM1_ESM.docx]

Supplementary Table 1. Geographical and economic information about the hospitals included for study

| Hospital | Hospital level | Geographical Region | 2016 GDP per capita（Dollar） | Eligible  patients | included  patients |
| --- | --- | --- | --- | --- | --- |
| Guangdong Provincial People’s Hospital | Tertiary | South | 20,872.50 | 461,486 | 399,520 |
| Chongzuo People's Hospital | Tertiary | South | 5,464.85 | 126,351 | 81,233 |
| Wuhua People's Hospital | Secondary | South | 1,902.50 | 72,506 | 20,278 |
| The Ninth People's Hospital Of Chongqing | Tertiary | Southwest | 8,515.00 | 231,567 | 139,115 |
| Sichuan Provincial People's Hospital | Tertiary | Southwest | 11,317.65 | 554,623 | 412,426 |
| Zhejiang Provincial People's Hospital | Tertiary | East | 17,852.06 | 219,680 | 45,903 |
| Shanghai Ninth People's Hospital | Tertiary | East | 17,141.47 | 114,994 | 55,603 |
| The Second Hospital of Anhui Medical University | Tertiary | East | 11,784.71 | 315,014 | 236,771 |
| Xinjiang Uygur Autonomous Region People's Hospital | Tertiary | Northwest | 10,230.15 | 60,063 | 45,799 |
| The Second Affiliated Hospital of Jilin University | Tertiary | North | 11,568.68 | 135,834 | 114,288 |
| Dongguan People's Hospital | Tertiary | South | 12,159.12 | 441,049 | 291,679 |
| Inner Mongolia Autonomous Region People's Hospital | Tertiary | North | 15,181.62 | 314,062 | 200,213 |
| The First People's Hospital of Kashgar | Secondary | Northwest | 2,479.41 | 310,707 | 199,871 |
| Guangdong Lufeng People's Hospital | Secondary | South | 2,722.50 | 131,961 | 105,340 |
| Huhhot First Hospital | Tertiary | Notth | 15,181.62 | 126,581 | 116,356 |
