# Supplementary material for: Feasibility of coding-based Charlson comorbidity index for hospitalized patients in China, a representative developing country
Source: BMC Health Serv Res. 2020 May 18;20:432. doi: 10.1186/s12913-020-05273-8 (PMC7236530; doi:10.1186/s12913-020-05273-8)
Supplement: Supplementary file 2 — Additional file 2. Table S2. Previously reported ICD-10 coding for Charlson comorbidity index. [file 12913_2020_5273_MOESM2_ESM.docx]

Supplementary Table 2. Previously reported ICD-10 coding for Charlson comorbidity index

| Disease | Score | ICD-10 code |
| --- | --- | --- |
| Myocardial infarction | 1 | I21; I22; I23; I25.2; I25.3; |
| Congestive heart failure | 1 | I50; I11.0; I13.0; I13.2; I25.5; |
| Peripheral vascular disease | 1 | I70; I71; I72; I73; I74; I731; I738; I739; I77; I771; I790; I792; K551; K558; K559; Z958; Z959; R02; K55.003; K55.004; K55.010; |
| Cerebrovascular disease | 1 | I60; I61; I62; I63; I65; I66; G450; G451; G452; G458; G459; G46; I64; G454; I670; I671; I672; I674; I675; I676; I677; I678; I679; I681; I682; I688; I69; I60-I69; G45; G46; |
| Dementia | 1 | F00; F01; F02; F03; F051; G30; G311; |
| Chronic pulmonary disease | 1 | J40; J41; J42; J44; J43; J45; J46; J47; J67; J60; J61; J62; J63; J66; J64; J65; I278; I279; J684; J701; J703; J84.1; J92.0; J96.1; J98.2; J98.3; J84.9; |
| Connective tissue disease | 1 | M32; M34; M332; M331; M053; M058; M059; M060; M063; M069; M050; M052; M051; M353; M05; M06; M315; M33; M351; M360; M08; M09; M30; M31; M35; M36; D86; |
| Ulcer disease | 1 | K25; K26; K27; K28; K22.1; |
| Mild liver disease | 1 | B18; K700-K703; K709; K713; K715; K717; K73; K74; K760; K762-K764; K768; K769; Z944; K71.0; K71.6; K71.9; K75; K76.103; B16.9; B19.9; |
| Diabetes mellitus | 1 | E109; E119; E139; E149; E101; E111; E131; E141; E135; E100; E110; E11.600; E120; E121; E126; E128-E131; E136; E138-E141; E148; E149; E11.701; E146 |
| Hemiplegia | 2 | G81; G041; G820; G821; G822; G114; G801; G802; G82; G830; G831-G834; G839; G838; |
| Moderate/severe renal disease | 2 | N03; N052; N053; N054; N055; N056; N072; N073; N074; N01; N18; N19; N25; I120; I131; N057; Z490; Z491; Z492; Z940; Z992/I12; I13; N00-N05; N07; N11; N14; N17; Q61; |
| Diabetes mellitus with chronic complications | 2 | E102; E112; E132; E142; E103; E113; E133; E143; E104; E114; E134; E144; E105; E107; E115; E122-E125; E127; E135; E137; E145; E147; E10.6; E10.8; E11.601; E11.8; E11.6; E14.5; |
| Any tumor | 2 | C00-C76; |
| Leukemia | 2 | C91-C95; C91.001; C95.902; |
| Lymphoma | 2 | C81-C85; C88; C90; C96; |
| Moderate/severe liver disease | 3 | K729; K766; K767; K721; I850; I859; I864; I982; K704; K711; K765; B15.0; B15.9; B16.0; B16.2; B19.0; K72; I85; Z944; |
| Metastatic solid tumor | 6 | C77-C80; |
| AIDS | 6 | B20; B21; B22; B23; B24; |
