# Supplementary material for: Feasibility of coding-based Charlson comorbidity index for hospitalized patients in China, a representative developing country
Source: BMC Health Serv Res. 2020 May 18;20:432. doi: 10.1186/s12913-020-05273-8 (PMC7236530; doi:10.1186/s12913-020-05273-8)
Supplement: Supplementary file 3 — Additional file 3. Table S3. Prevalence of comorbidities based on ICD-10 Coding Algorithms and diagnosis at different hospital levels. [file 12913_2020_5273_MOESM3_ESM.docx]

Supplementary Table 3. Prevalence of comorbidities based on ICD-10 Coding Algorithms and diagnosis at different hospital levels

|  | Tertiary hospital | |  | Secondary hospital | |  | Total | |
| --- | --- | --- | --- | --- | --- | --- | --- | --- |
| Comorbidities | ICD-based (%) | Diagnosis- based (%) |  | ICD-based (%) | Diagnosis- based (%) |  | ICD- based (%) | Diagnosis- based (%) |
| Myocardial infarction | 1.8 | 2.5 |  | 2.4 | 2.4 |  | 1.8 | 2.5 |
| Congestive heart failure | 6.9 | 10.2 |  | 11.1 | 10.2 |  | 7.5 | 10.2 |
| Peripheral vascular disease | 7.7 | 12.4 |  | 1.2 | 12 |  | 6.9 | 12.4 |
| Cerebrovascular disease | 12.2 | 11.7 |  | 12.6 | 12.3 |  | 12.2 | 11.8 |
| Dementia | 0.5 | 0.5 |  | 0.5 | 0.7 |  | 0.5 | 0.5 |
| Chronic pulmonary disease | 6.8 | 7.4 |  | 9.2 | 11.5 |  | 7.1 | 8.0 |
| Rheumatologic disease | 1.7 | 2.6 |  | 0.5 | 0.6 |  | 1.5 | 2.3 |
| Peptic ulcer disease | 1.3 | 1.7 |  | 1.2 | 1.2 |  | 1.3 | 1.7 |
| Mild liver disease | 9.3 | 13.1 |  | 5.7 | 3.7 |  | 8.8 | 11.8 |
| Diabetes without chronic complication | 8.8 | 9.0 |  | 7.8 | 8.0 |  | 8.7 | 8.9 |
| Hemiplegia | 0.2 | 1.0 |  | 0.2 | 0.7 |  | 0.2 | 0.9 |
| Renal disease | 6.3 | 7.6 |  | 3.6 | 3.5 |  | 5.9 | 7.1 |
| Diabetes with chronic complication | 2.8 | 3.0 |  | 2.7 | 2.6 |  | 2.8 | 2.9 |
| Tumor | 10.1 | 12.2 |  | 3.6 | 8.6 |  | 9.2 | 11.7 |
| Leukemia | 0.7 | 0.4 |  | 1.1 | 0.4 |  | 0.6 | 1.0 |
| Lymphoma | 0.9 | 1.0 |  | 0.2 | 0.3 |  | 0.8 | 0.9 |
| Moderate or severe liver disease | 0.7 | 1.1 |  | 0.3 | 0.4 |  | 0.6 | 1.0 |
| Metastatic solid tumor | 3.9 | 5.7 |  | 1.5 | 1.6 |  | 3.6 | 5.2 |
| AIDS | 0.1 | 0.1 |  | 0.2 | 0.2 |  | 0.1 | 0.1 |
