# Supplementary material for: Feasibility of coding-based Charlson comorbidity index for hospitalized patients in China, a representative developing country
Source: BMC Health Serv Res. 2020 May 18;20:432. doi: 10.1186/s12913-020-05273-8 (PMC7236530; doi:10.1186/s12913-020-05273-8)
Supplement: Supplementary file 4 — Additional file 4. Table S4. AUC for in-hospital mortality using ICD-based and diagnosis-based CCI. [file 12913_2020_5273_MOESM4_ESM.docx]

Supplementary Table 4. AUC for in-hospital mortality using ICD-based and diagnosis-based CCI

| Hospital | ICD-based CCI | Diagnosis-based CCI | *P* |
| --- | --- | --- | --- |
| 1 | 0.729 (0.720, 0.738) | 0.735 (0.725, 0.744) | 0.002 |
| 2 | 0.672 (0.610, 0.734) | 0.733 (0.678, 0.788) | 0.033 |
| 3 | 0.739 (0.716, 0.761) | 0.717 (0.694, 0.740) | 0.011 |
| 4 | 0.603 (0.582, 0.625) | 0.585 (0.562, 0.608) | 0.013 |
| 5 | 0.556 (0.516, 0.596) | 0.590 (0.548, 0.631) | 0.009 |
| 6 | 0.670 (0.652, 0.689) | 0.657 (0.638, 0.675) | <0.001 |
| 7 | 0.660 (0.642, 0.678) | 0.700 (0.681, 0.718) | <0.001 |
| 9 | 0.774 (0.768, 0.781) | 0.814 (0.808, 0.819) | <0.001 |
| 10 | 0.586 (0.549, 0.623) | 0.653 (0.618, 0.689) | <0.001 |
| 11 | 0.726 (0.702, 0.750) | 0.735 (0.713, 0.762) | 0.001 |
| 14 | 0.713 (0.700, 0.725) | 0.718 (0.705, 0.730) | 0.234 |
| 15 | 0.843 (0.819, 0.868) | 0.849 (0.817, 0.865) | 0.625 |
| 16 | 0.763 (0.748, 0.777) | 0.776 (0.762, 0.790) | 0.010 |
| 17 | 0.668 (0.657, 0.679) | 0.689 (0.678, 0.700) | <0.001 |
| 21 | 0.765 (0.757, 0.774) | 0.799 (0.791, 0.806) | <0.001 |
